# Evidence for lateralization of fear emotions in the cerebellum

**DOI:** 10.1007/s00415-025-13183-0

**Published:** 2025-05-30

**Authors:** Johanna Pakusch, Enzo Nio, Thomas Grosch, Thomas M. Ernst, Giorgi Batsikadze, Onur Güntürkün, Dagmar Timmann, Melanie D. Mark

**Affiliations:** 1 Behavioral Neuroscience, Ruhr-University Bochum, Universitätsstraße 150, ND7/33, 44780 Bochum, Germany; 2 Department of Neurology and Center for Translational Neuro- and Behavioral Sciences (C-TNBS), Essen University Hospital, Essen, Germany; 3 Erwin L. Hahn Institute for Magnetic Resonance Imaging, University of Duisburg-Essen, Essen, Germany; 4 Biopsychology, Faculty of Psychology, Ruhr-University Bochum, 44780 Bochum, Germany

Dear Sirs,

Hemispheric specialization is a well-established principle in neuroscience, with the cerebral cortex showing distinct lateralization for cognitive and emotional processes. It is likely that asymmetries serve at least two purposes. First, by specializing on one limb or one sensory side, the contralateral hemisphere goes through life-long cycles of motor and perceptual learning, thereby increasing processing speed and motor efficacy, while decreasing reaction time. Second, complementary specialized hemispheres allow parallel neural processes of task components, thereby reducing redundancy [6]. Although there is compelling evidence in humans and mice for the lateralization of emotions in the cerebral cortex, very little evidence for lateralization of emotions within the cerebellum is available.

Since the cerebellar cortex is contralaterally connected to the cerebral cortex, each hemisphere of the cerebellum primarily interacts with the opposite hemisphere of the cerebrum. Therefore, cognitive and emotional functions such as language and negative emotions, which are strongly associated with right hemispheric processing in the cerebrum, would be expected to be more influenced by the left cerebellar hemisphere [8].

In a recent review [11], the authors highlight the right cerebellar lateralization for language processing, while other cognitive and affective functions exhibit less defined lateralizations. The foundation of the present study is an incidental observation  from four independent 7-Tesla fMRI fear conditioning studies in humans previously performed by our group, in which we paired a visual conditioned stimulus (CS) with an electrical shock as the unconditioned stimulus (US). Fear conditioning is a widely used paradigm for studying emotional regulation, particularly the ability to adapt emotional responses to changes in the environment [9]. For example, it is crucial for survival to learn that certain stimuli predict the occurrence of dangerous situations. Furthermore, it is highly adaptive to respond with fear to these predictors, thereby avoiding potential threats. Equally important is the ability to unlearn these associations when they are no longer relevant. A failure to extinguish fear associations is thought to be a key factor in the development of anxiety disorders.

In our fear conditioning studies, we consistently observed predominantly left-sided activation of the cerebellum when the US was unexpectedly omitted in unreinforced acquisition and early extinction trials (Fig. 1; [2, 3, 5, 12]). The unexpected US omission is thought to represent prediction errors driving extinction learning [5, 12]. Importantly, the predominantly left-sided activation was not influenced by whether the shock was applied to the left or right limb (hand or shin), indicating that cerebellar lateralization was not simply a consequence of sensory input location. We conducted a statistical comparison of beta values within a cerebellar volume-of-interest, which was defined by a conjunction analysis of contrasts related to unexpected US omission in our most recent dataset [12]. This region primarily encompassed lobule VI and Crus I and revealed significantly higher beta values (*p* = 0.003) in the left cerebellar hemisphere compared to the right. These findings further suggest the presence of cerebellar lateralization in emotional processing independently of stimulus location.

**Fig. 1:**
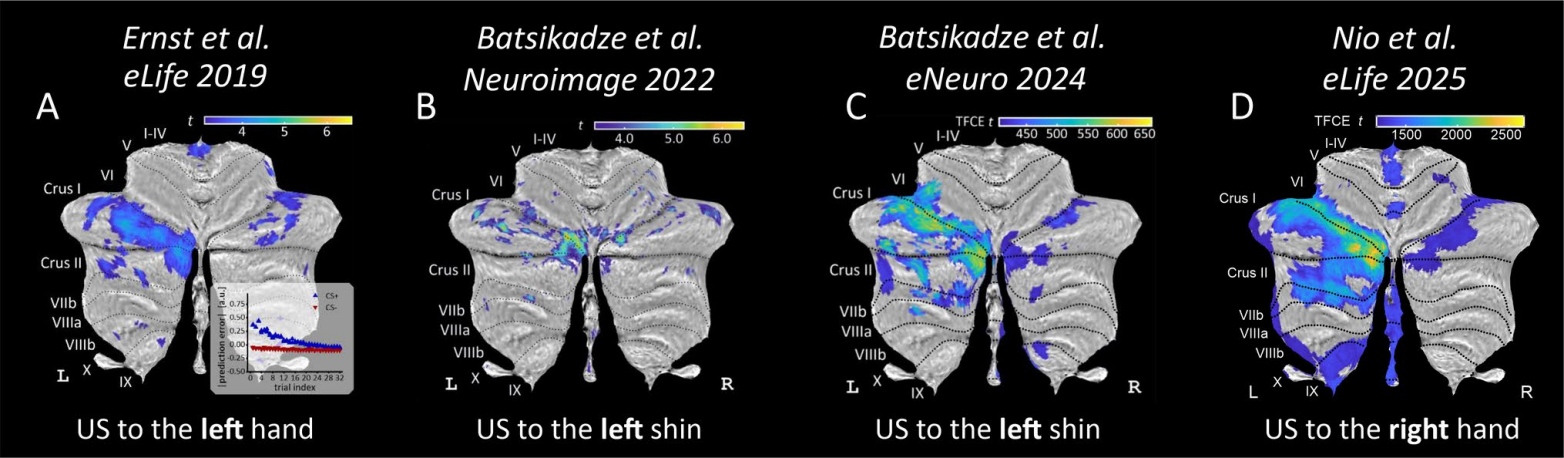
Human cerebellar activation during unexpected US omissions in extinction, visualized on cerebellar flatmap projections (SUIT; [4]), was predominantly left-lateralized in lobule VI, Crus I, and Crus II, likely reflecting its role in processing prediction errors during extinction learning. Color maps indicate statistical significance (*t*-values or threshold-free cluster enhancemed (TFCE) *t*-values; **A**–**C** uncorrected; **D** FWE-corrected; all *p* < 0.05). **A** Reanalyzed data from [5], eLife) during unexpected US omissions in the extinction phase showed a prediction error signal driving left-sided activation. Inset: Prediction error plot for CS + (conditioned stimulus paired with US) and CS- trials over time. **B** [2], Neuroimage) applied US stimulation to the left shin. During extinction, unexpected US omissions modulated with estimated prediction errors revealed left-sided activations. **C** [3], eNeuro) contrasted unexpected US omissions in early extinction (left shin stimulation) against rest to show left-sided activation. **D** [12], eLife) found that, despite the change of US stimulation side to the right, activations in extinction driven by prediction errors during US omissions remained predominantly left-sided. (The four manuscripts are under creative commons license)

To investigate this lateralization effect, we observed in humans in greater detail, we monitored *c-fos* activity, a well-established marker of neuronal activity, which has been used extensively to unravel the regions and connections involved in fear learning and extinction. The targeted recombination in active populations (TRAP) system builds on *c-fos* to selectively and permanently label neurons that are active within a specific time window [7]. This two-component TRAP system first exploits the *c-fos* promoter to control the expression of the cre recombinase fused to the hormone-binding domain of the estrogen receptor (creER) in active neurons (Fig. 2). Second, upon binding to an estrogen ligand, 4-hydroxy-tamoxifen (4-OHT) is creER able to enter the nucleus, excise out the stop codon, and allow expression of the fluorescent marker protein tdTomato, providing us temporal control over the labeling of *c-fos* active neurons. Since *c-fos* expression can be detected 30–120 min after activity and 4-OHT levels in the brain exhibit rapid absorption with a peak at 1 h after injection, we timed the 4-OHT injection to approximately align with the expected peak in *c-fos* promoter activity. Specifically, TRAP mice were injected with 4-OHT 30 min after the early extinction session to permanently label neurons activated during fear extinction learning.

**Fig. 2:**
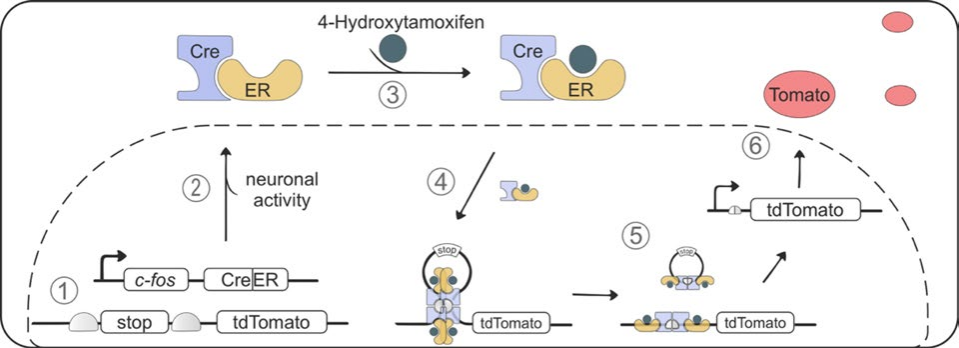
Schematic of targeted recombination in active populations (TRAP). Mechanism how *c-fos* induced neuronal activity in the cerebellum is labeled by the fluorescent protein tdTomato. **1** Under basal conditions, the *c-fos* promoter is inactive and cre recombinase fused to the estrogen receptor (creER) is not expressed. tdTomato is also not expressed due to the stop codon. **2** The *c-fos* promoter is stimulated in active neurons, leading to creER expression in the cytosol. **3** Administration of 4-hydroxytamoxifen binds to creER and subsequent **4** translocation into the nucleus. **5** creER forms a tetramer which interacts with loxP sites that flank the stop codon and excise the stop codon, thus allowing the expression of tdTomato in *c-fos* active neurons. *Cre* cre recombinase; *ER* estrogen receptor; *Fos* *c-fos* promoter

Targeted recombination in active populations of mice underwent a cue-dependent fear conditioning paradigm analogous to the one used in our human studies. During acquisition, a tone (CS) was paired with a shock (US), leading to increasing conditioned responses (CR, freezing). Twenty-four hours later, mice were assigned to either a fear extinction (FE) group which received repeated CS in a new context but no US, or a novel context (no extinction, NE) control group without CS or US exposure (Fig. 3A). During early extinction and retrieval, augmented fear responses were demonstrated in the FE group where the US was omitted but not in controls where both CS and US were removed (Fig. 3B1 and B2, Supplementary Table 2). To label neurons engaged during extinction learning, all animals were injected with 4-OHT 30 min after the first extinction session. This activates the TRAP system, which permanently tags *c-fos* active neurons (a proxy for neural activity) within a 2 h time window [7]. After 3 days of extinction training, a CS was presented alone to test if the mice could recall the previous association of the CS to US with a CR. The FE mice extinguished their previous association of the CS to US due to the repetitive CS exposures without the US (extinction) which resulted in a reduction of CRs (Fig. 3B2). While the NE group retained a heightened freezing response, since they were not exposed to the CS during extinction (Fig. 3B2).

**Fig. 3:**
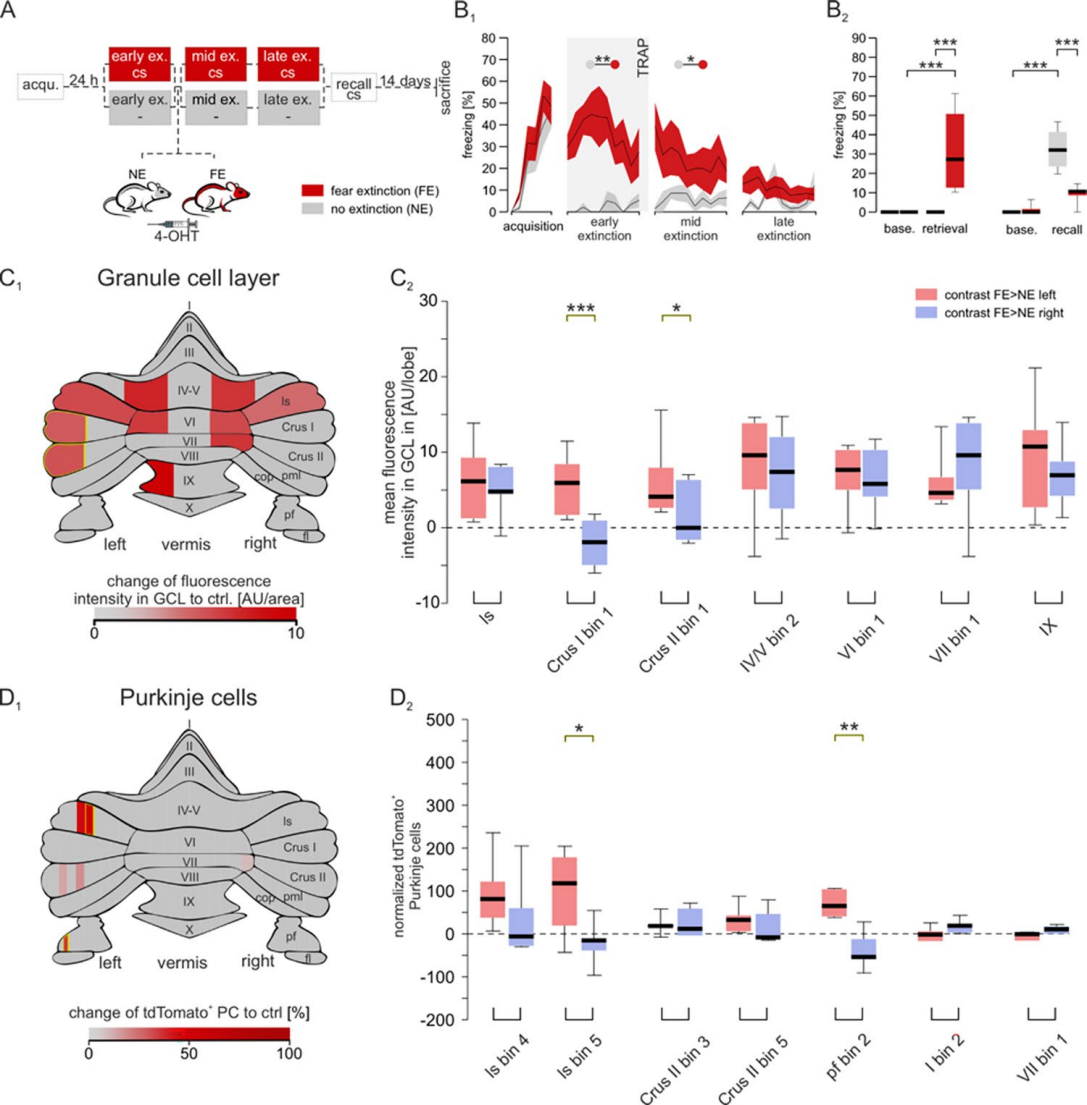
Granule cell layer and Purkinje cell activity across different cerebellar subregions during early extinction. **A** Schematic of the experimental design. Mice initially underwent fear acquisition (acqu.). After 24 h, mice were divided into two groups; the red group underwent extinction (ex.) training (FE, fear extinction), while the grey group was not subjected to the tone (NE, no extinction). Following extinction, all animals were injected with 4-OHT. A total of 3 extinction days was conducted before extinction recall, where both groups were subjected to the CS. **B** Freezing behavior during the fear conditioning paradigm **B**_**1**_ Time-course traces show the freezing behavior during the CS for both groups. During the acquisition, both groups displayed increasing freezing behavior (F(3.347,34.74) = 22.41, *p* =  < .001), while extinction elicited higher freezing in the FE group (F(1,13) = 17.04, *p* = .001). **B**_**2**_ Boxplots display the freezing responses during baseline and retrieval between groups, as well as the difference between baseline and recall with statistical significance marked with asterisks. Fear recall revealed reduced fear behavior in the FE group when compared to the NE (post-hoc Tukey *p* =  < .001). Shaded areas represent SEMs, with significant differences indicated by asterisks which were determined with a two-way Mixed effects analysis with Greenhouse Geisser correction (*p* > 0.05). **C**_**1**_ Heatmap of mean fluorescent signals in the granule cell layer (corrected to controls) divided into examined areas. The gradient spans from control group levels to above control levels in red. Grey areas represent areas which did not reach significance. **C**_**2**_ Boxplots of granule cell layer activity in subregions displaying significant differences between left (light red) and right (light blue) hemispheres. Increased granule cell layer activity was observed in the left hemisphere for Crus I (t(6) = 7.01, *p* =  < .001) and Crus II (t(6) = 2.942, *p* = .026). **D**_**1**_ Heatmap of normalized mean fluorescent signals in Purkinje cells (corrected to controls) divided into 10% bins. The gradient spans from control group levels to above control levels in red. Grey areas represent areas which did not reach significance. **D**_**2**_ Boxplots of Purkinje cell activity in subregions displaying significant differences between left (light red) and right (light blue) hemispheres. Increased Purkinje cell activity was observed in the left hemisphere for lobule simplex (t(6) = 3.306, *p* = .015) and paraflocculus (t(5) = 5.1, *p* = .004). Statistical testing for hemispheric differences was analyzed with multiple paired t-tests as implemented in GraphPad Prism. All data are reported in the Supplementary Tables. Schematic of the cerebellar flatmap was adapted from [13]. *ls* lobule simplex; *pml* paramedian lobule; *cop* copula pyramidis; *pf* paraflocculus; *fl* flocculus

*C-fos* induced neuronal activity from granule cell layer, and Purkinje cells were then quantified at early extinction from TRAP mouse cerebella, since increased cerebellar activity in the left-sided hemisphere was observed in our human fMRI studies during early extinction or when the US was unexpectedly omitted. Since human fMRI signals are influenced primarily by granule cell (GC) activity and synaptic input from mossy fibers [10], we first measured changes in *c-fos*-induced fluorescent intensities from distinct GC layer subregions during early extinction when the US was unexpectedly omitted by comparing normalized GC layer fluorescent intensities in the FE to NE group per bin of each lobule. In line with the human findings, we found greater GC layer activity changes in the left hemisphere of the cerebellum during early extinction. More specifically, we found increased GC layer activity in subregions of vermal lobules IV/V, VI, VII and IX (Fig. 3C1, Supplementary Table 4), as well as in hemispheric subregions of lobule simplex (hemispheric VI in humans), Crus I and Crus II. After comparing the changes in GC layer activity between left and right hemispheres, we measured higher rises in the left-sided hemispheric subregions of Crus I (*p* =  < 0.001) and Crus II (*p* =  < 0.026) during early extinction when the US was unexpectedly omitted (Fig. 3C2, Supplementary Table 5). These results agree with our fMRI studies in humans where a left-sided hemispheric dominance in Crus I was also observed when the US was unexpectedly omitted. To gain deeper insights into the lateralized cerebellar activity across specific neuronal subpopulations, we additionally examined dynamic changes in the activity of Purkinje cells (PCs), the main output neuron of the cerebellum. They integrate diverse synaptic inputs and control the information to downstream structures, making them crucial to understand brain wide lateralized modulation. To identify differences in PC activity during early extinction, we compared the normalized PC activity in the FE to NE group per 10% bin of each lobule (Fig. 3D1). We detected augmented PC activity in right-lateralized subregions of lobules I and VII, but overall PC activity was more pronounced in the left hemispheric areas of the paraflocculus, Crus II and lobule simplex (Fig. 3D1, Supplementary Table 6). Importantly, comparisons of PC activity between the left and right hemispheric subregions demonstrated a left-sided dominance in PC activity in lobule simplex (*p* = 0.016) and paraflocculus (*p* = 0.004) (Fig. 3D2, Supplementary Table 7), further supporting a cerebellar lateralization of fear emotions that appears to be preserved across species. Together, our TRAP studies in mice highlight the importance of enhanced GC layer and PC neuronal activity in localized subregions of the lobule simplex (hemispheric VI in humans), Crus I and Crus II in the left hemisphere to be potentially contributing to the cerebellar lateralization of fear emotions.

Notably, our TRAP results agree with the observations in our human fMRI studies. Given the predominantly contralateral connectivity of the cerebellum to cortical areas, our findings support a lateralized role of the cerebellum in fear extinction, with predominantly left hemispheric activations of subregions. We found significantly greater beta values in the left cerebellar cortex when comparing to the right cerebellar hemisphere in humans. This is in agreement with past neuroimaging studies implicating a preferential role of the cerebellum in processing negative emotions and suggests a left-sided cerebellar dominance during fear processing. In addition, past clinical studies in stroke patients with left cerebellar lesions exhibited stronger impairments in distinguishing emotions related to aversive stimuli [1]. Moreover, Crus I in the left cerebellum was predominately activated during emotional processes in a fMRI meta-analysis [14], likely reflecting its connections to key emotional networks in the right cerebral hemisphere. Until now, the cerebellar hemispheric differences for fear conditioning paradigms were not analyzed. In our study, we report a cerebellar left hemispheric dominance of fear regardless whether the stimulation was applied to the left or right side of the body. This suggests that the observed asymmetry is not a consequence of the stimulation site but rather reflects an intrinsic lateralization of cerebellar processing during fear extinction.

Given the contralateral connectivity patterns of the cerebellar and the cortical cortices, our data accord with a right cortical hemispheric superiority in aversive emotional processing. Currently, three different theories on cortical emotional asymmetry make different predictions for positively valenced conditions, but agree that negatively valenced, fear-related processes are primarily processed by the right cortical hemisphere in humans and mice [6]. Taken together, all this supports the hypothesis, which lateralized cerebellar circuits modulate extinction of learned fear responses with a left-sided prevalence in regions associated with emotion regulation and memory updating. Flatmap representations display more pronounced activity in the left hemisphere with pronounced activations in lobule VI (lobule simplex in mice), Crus I and Crus II. Our findings provide further evidence that the cerebellum contributes to emotional regulation like fear extinction through hemispheric specialization, mirroring the lateralization observed in cortical fear-processing networks.

## Supplementary Information

Below is the link to the electronic supplementary material.

Supplementary file1 (DOCX 93 KB) We corrected the Supplementary file1 and sent it via email.

## Data Availability

All data will be made available by Melanie D. Mark (melanie.mark@rub.de) for the mouse studies and Dagmar Timmann for the human studies upon reasonable request.
