# Supplementary material for: Evidence for lateralization of fear emotions in the cerebellum
Source: J Neurol. 2025 May 30;272(6):435. doi: 10.1007/s00415-025-13183-0 (PMC12125079; doi:10.1007/s00415-025-13183-0)
Supplement: Supplementary file 1 — Supplementary file1 (DOCX 93 KB) We corrected the Supplementary file1 and sent it via email. [file 415_2025_13183_MOESM1_ESM.docx]

**Supplementary material**

**Material and Methods**

**Animals**

The local ethics committee (Bezirksamt Arnsberg) and the animal care committee of Nordrhein-Westfalen (LANUV; Landesamt für Umweltschutz, Naturschutz und Verbraucherschutz Nordrhein-Westfalen, Germany) approved all experiments. Studies were carried out using Fos^CreERT2 (+/−)^ × B6.Cg-Gt(ROSA)26Sor^tm9(CAG-tdTomato)Hze^/J ^(+/−)^ mice, further refered to as TRAP. TRAP mice were obtained by crossing Fos^CreER (+/−)^(RRID:IMSR_JAX:021882) and B6.Cg-Gt(ROSA)26Sor^tm9(CAG-tdTomato)Hze^/J ^(+/+)^ (RRID:IMSR_JAX:007909) mice. Animals were kept in groups of 2–3 with unlimited access to food and water. Prior to behavioral testing, mice were kept in a separate room with a 12 h light/dark cycle. All tests were conducted during the light phase. Fear conditioning was performed in mice of both sexes at 4 to 5 months of age. Mice were habituated to handling prior to behavioral testing.

**Drug preparation**

4-OHT (Sigma-Aldrich) was dissolved in DMSO and frozen. Shortly before use, 4-OHT was diluted with an 8% Tween 80/ saline solution. The final solution was 40 mg/kg 4-OHT and administered intraperitoneally (i.p.) to TRAP mice.

**Fear conditioning and TRAPing**

Cue fear conditioning was conducted using an AB context design. The contexts differed in terms of lighting, scent texture, and appearance. The conditioning chamber (23 × 25 × 24 cm) was placed inside a noise-reducing cabinet. A centrally mounted speaker (FR 58 VISATON) delivered the conditioned stimulus (CS). The animals were video recorded (Mako U-130B Allied Vision Technologies) to enable post-hoc analysis of fear behavior. A custom MATLAB (The MathWorks) script controlled the timing of tone, shock and video recording.

To investigate active neurons during fear extinction, mice underwent fear acquisition (FC) in context A where the mice had a 2 min baseline period, followed by 6 tone/shock pairings (CS 30 s, 7.5 kHz, 60 dB/us 2 s 0.45 mA co-terminating with the CS). The inter-trial-interval (ITI) ranged from 60 to 180 s. The chamber was thoroughly cleaned between animals. 24 h later, the mice were brought to context B for fear extinction. The control group was exposed to the extinction context without CS presentation (NE). The test group underwent extinction training (FE) starting with a 2 min baseline followed by 10 CS presentations (CS 30 s, 7.5 kHz, 60 dB). The ITIs varied between 30 s and 180 s. 30 min after the start of early extinction. The mice were injected i.p. with 4-OHT and returned to their home cage. Extinction was repeated twice (mid and late extinction), but without 4-OHT injection. On day four, both groups underwent recall of extinction with exposure to the CS, to ensure that the control group did express fear behavior to the CS and the extinction group showed adequate extinction learning.

**Fear analysis**

EthoVision XT 11.5 (Noldus Information Technology) was used to analyze freezing behavior as a readout of fear. To analyze freezing, the changing pixels from one frame to the next were set to a threshold to fulfill the criteria of freezing, which is the absence of movement except for respiratory movement for two consecutive seconds. The automated analysis was verified manually. Freezing was analyzed during 30 s CS presentation and baseline activity before CS presentation.

**Histological analysis and statistical testing**

Two weeks following the behavior, mice were anesthetized with ketamine/xylazine (100/10 mg/kg) and transcardially perfused with phosphate buffered saline (PBS) followed by 4% paraformaldehyde in PBS (PFA). The brains were post-fixed for 4-6 h in PFA and then transferred into 30% sucrose for at least 48 h. Brains were subsequently embedded in Tissue-Tek O.C.T. compound (optimal cutting temperature; Sakura) and 40 µm sagittal cryo-sections (Leica CM3050S) were obtained. Sections were mounted with Mowiol DABCO and granule cell layer images were acquired using a confocal microscope (Leica Microsystems TCS SP5II). The number of Purkinje cells was determined via live imaging and identified by their morphological properties as well as their localization within the cerebellum (Leica M205 FCA).

GraphPad Prism (GraphPad Software, San Diego, California, USA, [www.graphpad.com](http://www.graphpad.com)) was used for data visualization and post-processing using CorelDraw® Graphics Suite (Corel Corporation, Ottawa, Canada). Fear behavior is plotted as the mean ± SEM (shaded area). Fear behavior was analyzed using two-way repeated-measures mixed-effects analysis (two-way RM MEA with Geisser-Greenhouse correction (GGC)), as implemented in GraphPad. RM MEA was used to analyze changes in freezing behavior over the course of the trial and between groups, as well as the interaction between groups and trials. Differences in freezing between groups during baseline, retrieval and recall were plotted as boxplots with whiskers representing 10-90 percentiles and were statistically analyzed using two-way RM MEA with GGC, followed by post-hoc Tukey´s multiple comparison test. Neuronal activity was analyzed using two-way RM MEA with GGC with post-hoc multiple comparison (Fisher LSD) between groups per lobule.

PC numbers were counted per lobule in each 40 µm sagittal brain section. For graphical visualization. PC numbers were calculated for each brain and each lobule per 10% section and subsequently normalized for each brain (0% being the smallest PC number, 100% the average PC number in a particular brain area). We plotted the differences (FE-NE) between the groups in the flatmap. To plot the PC numbers of PCs, we normalized as stated above for each lobule and plotted them as boxplots with whiskers representing the 10-90 percentiles. GCL analysis was performed at seven locations throughout the cerebellar cortex. Each location was analyzed using a set of two images of the respective location. The locations are oriented at the individual brain, coordinates serve as orientation (1/7: center of the flocculus ML: 3.1; 2/6: center of copula pyramidis ML: 2.04; 3/5: first/ last quarter of VIII ML: 0.9; 4: center of lobule I ML: 0. Fiji (Schindelin et al., 2012) was used to analyze the GCL by manually setting the ROIs around each GCL per lobule. The mean intensity per ROI. We plotted the intensity of fluorescence as the differences between the groups graphically in a heatmap manner. The GCL intensity were plotted as boxplots with whiskers representing 10-90 percentiles

**Humans**

**Paradigm overview**

We conducted four differential fear conditioning studies to explore fear conditioning, presented in order of publication:

- **Ernst et al. (2019)**: A 1-day paradigm combining acquisition and extinction in a single session.
- **Batsikadze et al. (2022)**: A 2-day paradigm with acquisition and extinction on Day 1 and a recall test on Day 2.
- **Batsikadze et al. (2024)**: A 2-day paradigm with acquisition and extinction on Day 1 and recall on Day 2.
- **Nio et al. (2025)**: A 3-day paradigm with acquisition on Day 1, extinction on Day 2, and recall/reacquisition assessed on Day 3.

All paradigms featured a conditioned stimulus (CS+) paired with an unconditioned stimulus (US; electric shock) and an unpaired CS- (never reinforced). All data were previously published.

**Stimuli and timing**

Visual CSs varied across studies:

- In **Ernst et al. (2019)** and **Nio et al. (2025)**, CSs were geometric shapes (square and diamond).

In **Batsikadze et al. (2022, 2024)**, CSs were a colored lamp light signaling the CS type. The CS was presented for 6-8 seconds, with the US (a 0.1-second electric shock) delivered at the end of reinforced CS+ trials. Supplementary Table 1 summarizes key paradigm details, including CS length and reinforcement rates for CS+ trials.

Supplementary Table 1: Summary of human fear conditioning paradigms

| **Study** | **Year** | **Days** | **CS Type** | **CS Length** | **Reinforcement Rate (CS+)** | **Extinction timing** | **Additional Notes** |
| --- | --- | --- | --- | --- | --- | --- | --- |
| Ernst et al. | 2019 | 1 | Geometric shapes | 8 s | 62.5% | Day 1 | Re-analyzed with prediction errors |
| Batsikadze et al. | 2022 | 2 | Photos w/ context | 8 s | 62.5% | Day 1 | Unextinguished CS+U |
| Batsikadze et al. | 2024 | 2 | Photos w/ context | 8 s | 66.7% | Day 1 | - |
| Nio et al. | 2025 | 3 | Geometric shapes | 6 s | 100% | Day 2 | Recall/reacquisition (83.3%) on Day 3 |

**Behavioral and physiological measures**

Fear acquisition and extinction learning were assessed using skin conductance responses measured throughout experimental phases to track autonomic fear responses, alongside questionnaires administered after each phase to evaluate self-reports pertaining to arousal, fear, valence, and CS-US contingency awareness.

**FMRI Analysis**

FMRI data were collected and analyzed using an event-related design in SPM12 (Statistical Parametric Mapping), with events defined as CS onset and US presentation or omission (duration: 0 s). Preprocessing included normalization to Montreal Neurological Institute (MNI) space using the SUIT (Spatially Unbiased Infratentorial Template) toolbox and CAT12 (Computational Anatomy Toolbox) within SPM12.

Prediction error estimates were calculated using models detailed in Batsikadze et al. (2022, 2024) and Nio et al. (2025), modulating US omission contrasts in all studies except Batsikadze et al. (2024), where early US omissions were compared vs. rest. Ernst et al. (2019) results were based on a re-analysis with prediction errors, not included in the original paper.

In Nio et al. (2025), a cerebellar volume of interest (VOI; lobule VI and Crus I) was derived from a global conjunction analysis of prediction error modulation contrasts across extinction, recall, and reacquisition phases, masked to the cerebellum. The VOI was located on the left hemisphere and mirrored on the x-axis to produce a right-lateralized VOI. Mean beta values for the prediction error modulated US omission regressor were extracted separately from the left- and right-hemisphere VOIs and compared using a paired t-test. Significance was set at p < 0.05. For plotting of cerebellar flatmaps, Threshold-Free Cluster Enhancement (TFCE) was applied in Batsikadze et al. (2024) and Nio et al. (2025). Family-Wise Error (FWE) correction was used in Nio et al. (2025); the other studies used uncorrected thresholds.

**Nomenclature across humans and mice**

The nomenclature of cerebellar lobes differs between humans and mice. To ensure clarity and accessibility for all readers, we used the mouse-specific nomenclature when presenting mouse data, with the corresponding human lobe nomenclature provided in parentheses.

**Supplementary Tables**

Supplementary Table 2: TRAP during early extinction behavior

| ***FE/NE*** | **Factor** | **Num Df** | **Den Df** | ***F*** | ***p-value*** |
| --- | --- | --- | --- | --- | --- |
| **TRAP extinction MEA behavior** | | | | | |
| *fear acquisition training* | | | | | |
| 7/5 | Trial  Group  Trial × group | 3.474  1  5 | 4.74  0  0 | 22.41  0.7797  0.368 | **< .0001**  0.398  0.868 |
| *early extinction training* | | | | | |
| 7/5 | Trial  Group  Trial × group | 1.784  1  9 | 3.19  3  17 | 1.61  17.04  2.774 | 0.222  **0.001**  **0.006** |
| *mid extinction training* | | | | | |
| 7/5 | Trial  Group  Trial × group | 1.7  1  9 | 2.10  3  17 | 1.376  5.605  1.843 | 0.270  **0.034**  0.068 |
| *late extinction training* | | | | | |
| 7/5 | Trial  Group  Trial × group | 4.27  1  9 | 5.5  3  17 | 1.277  1.038  2.19 | 0.289  0.327  **0.027** |

Supplementary Table 3: TRAP during extinction behavior baseline, retrieval and recall

| ***FE/NE*** | | **Factor** | **Num Df** | **Den Df** | | | ***F*** | | ***p-value*** |  |
| --- | --- | --- | --- | --- | --- | --- | --- | --- | --- | --- |
|  | | | | | | | | | |  |
| 7/5 | Trial (Baseline x Retrieval x Baseline x Recall)  Group  Trial × Genotype | | 3  1  3 | | 30  10  30 | 8.98  0.870  21.53 | | **<.001**  0.373  **<.001** | | |
| **Post-hoc test (Turkey)**   \| **Group** \| **Trial** \| ***p-value*** \| \| --- \| --- \| --- \| \| FE. \| Baseline x Retrieval \| **<.001** \| \| NE \| Baseline x Retrieval \| >.999 \| \| FE \| Baseline x Recall \| 0.286 \| \| NE \| Baseline x Recall \| **<.001** \| \| FE x NE \| Baseline \| >.999 \| \| FE x NE \| Retrieval \| **<.001** \| \| FE x NE \| Baseline \| 0.818 \| \| FE x NE \| Recall \| **<.001** \| | | | | | | | | | |  |

Supplementary Table 4: Extinction evoked GCL activity changes on a sublobular level

| **Early extinction Fisher´s LSD GCL Intensity (Heatmap)** | | | | |
| --- | --- | --- | --- | --- |
| **Right/ left** | **lobule** | **mean diff.** | **95.00% CI of diff.** | **p-value** |
| right | FL | -2.331 | -9.899 to 5.236 | .503 |
|  | PF | 1.621 | -8.197 to 11.44 | .699 |
|  | PML lateral | 2.040 | -5.053 to 9.133 | .505 |
|  | PML vermal | 5.381 | -1.916 to 12.68 | .125 |
|  | CRUS I lateral | -2.240 | -15.34 to 10.86 | .670 |
|  | CRUS I vermal | 4.777 | -1.815 to 11.37 | .132 |
|  | CRUS II lateral | 1.734 | -4.845 to 8.313 | .553 |
|  | CRUS II vermal | 5.738 | -0.7947 to 12.27 | .076 |
|  | LS | 4.958 | 1.273 to 8.643 | **.014** |
|  | COP | 1.637 | -8.670 to 11.94 | .713 |
| vermal | X right | 8.568 | -0.3366 to 17.47 | .057 |
|  | X vermal | 6.324 | -0.1641 to 12.81 | .055 |
|  | X left | 6.176 | -1.786 to 14.14 | .110 |
|  | IX right | 6.896 | -10.31 to 24.11 | .340 |
|  | IX vermal | 8.003 | -5.776 to 21.78 | .200 |
|  | IX left | 9.923 | 0.4006 to 19.44 | **.043** |
|  | VIII right | 7.614 | -0.9115 to 16.14 | .070 |
|  | VIII vermal | 6.852 | -10.76 to 24.46 | .351 |
|  | VIII left | 6.365 | -5.053 to 17.78 | .227 |
|  | VII right | 7.421 | 0.6949 to 14.15 | **.035** |
|  | VII vermal | 5.939 | -5.230 to 17.11 | .224 |
|  | VII left | 5.806 | -1.742 to 13.35 | .108 |
|  | VI right | 6.424 | 0.9797 to 11.87 | **.026** |
|  | VI vermal | 7.933 | -4.005 to 19.87 | .145 |
|  | VI left | 7.145 | 1.536 to 12.75 | .**019** |
|  | IV/V right | 0.8404 | -5.457 to 7.138 | .757 |
|  | IV/V | 7.466 | 1.558 to 13.37 | **.018** |
|  | IV/V vermal | 5.940 | -4.454 to 16.33 | .212 |
|  | IV/V | 8.239 | 2.225 to 14.25 | **.014** |
|  | IV/V left | 5.711 | -4.676 to 16.10 | .213 |
|  | III right | 5.633 | -1.560 to 12.83 | .112 |
|  | III vermal | 1.072 | -9.076 to 11.22 | .815 |
|  | III left | 6.258 | -2.181 to 14.70 | .128 |
|  | II right | 5.696 | -1.639 to 13.03 | .111 |
|  | II vermal | 1.072 | -9.076 to 11.22 | .815 |
|  | II left | 5.572 | -2.851 to 13.99 | .170 |
|  | I | 7.012 | -2.482 to 16.50 | .119 |
| left | FL | 2.162 | -5.235 to 9.559 | .526 |
|  | PF | 4.610 | -2.391 to 11.61 | .167 |
|  | PML vermal | 4.792 | -1.477 to 11.06 | .106 |
|  | PML lateral | 3.807 | -1.027 to 8.640 | .110 |
|  | CRUS I vermal | 4.990 | -6.484 to 16.46 | .300 |
|  | CRUS I lateral | 5.669 | 0.1662 to 11.17 | .**045** |
|  | CRUS II vermal | 4.882 | -2.483 to 12.25 | .144 |
|  | CRUS II  lateral | 6.078 | 1.244 to 10.91 | .**019** |
|  | LS | 6.433 | 0.4354 to 12.43 | **.038** |
|  | COP | 2.847 | -11.14 to 16.83 | .612 |

Supplementary Table 5: Hemispheric differences in GCL activity in sublobular areas

| **Early extinction multiple paired t test hemispheric difference of normalized GCL** | | | | |
| --- | --- | --- | --- | --- |
| **lobule** | **% of lobule** | **mean diff.** | **t ratio** | **p-value** |
| LS |  | -1.476 | 0.8401 | .433 |
| CRUS I | Bin 1 | -7.908 | 7.006 | **<.001** |
| CRUS II | Bin1 | -4.343 | 2.942 | .**026** |
| IV/V | Bin 2 | -0.7728 | 0.5384 | .610 |
| VI | lateral | -0.7206 | 0.5170 | .624 |
| VII | lateral | 2.434 | 1.025 | .345 |
| IX | lateral | -3.026 | 1.459 | .195 |

Supplementary Table 6: TRAP during early extinction normalized PC activity

| **Early extinction Fisher´s LSD normalized PC (Heatmap)** | | | | |
| --- | --- | --- | --- | --- |
| **lobule** | **% of lobule** | **mean diff.** | **95.00% CI of diff.** | **p-value** |
| PF right  lateral-vermal | 10% | 11.65 | -87.40 to 110.7 | .787 |
|  | 20% | -37.22 | -110.7 to 36.23 | .265 |
|  | 30% | -12.60 | -86.12 to 60.91 | .674 |
|  | 40% | 9.700 | -70.89 to 90.29 | .785 |
|  | 50% | 4.269 | -48.31 to 56.85 | .857 |
|  | 60% | -17.05 | -73.99 to 39.89 | .514 |
|  | 70% | -9.526 | -49.90 to 30.85 | .581 |
|  | 80% | 60.92 | -45.78 to 167.6 | .231 |
|  | 90% | 16.62 | -115.5 to 148.8 | .785 |
|  | 100% | -49.22 | -238.2 to 139.8 | .560 |
| FL right  lateral-vermal | 10% | -31.73 | -125.3 to 61.84 | .411 |
|  | 20% | -25.22 | -118.9 to 68.49 | .536 |
|  | 30% | -75.16 | -185.9 to 35.57 | .157 |
|  | 40% | -69.79 | -218.7 to 79.09 | .310 |
|  | 50% | -67.23 | -251.8 to 117.4 | .408 |
|  | 60% | -5.831 | -126.4 to 114.7 | .914 |
|  | 70% | -7.186 | -181.9 to 167.5 | .927 |
|  | 80% | 66.23 | -95.24 to 227.7 | .381 |
|  | 90% | 7.891 | -161.6 to 177.4 | .914 |
|  | 100% | 19.59 | -114.5 to 153.7 | .750 |
| COP right  lateral-vermal | 10% | -22.38 | -63.22 to 18.45 | .228 |
|  | 20% | -13.88 | -47.73 to 19.98 | .373 |
|  | 30% | -16.97 | -41.01 to 7.075 | .146 |
|  | 40% | -28.12 | -61.61 to 5.381 | .091 |
|  | 50% | -8.956 | -55.86 to 37.94 | .654 |
|  | 60% | -6.047 | -33.65 to 21.55 | .633 |
|  | 70% | -1.783 | -44.90 to 41.34 | .926 |
|  | 80% | -7.131 | -43.46 to 29.20 | .668 |
|  | 90% | -19.19 | -53.20 to 14.82 | .228 |
|  | 100% | -4.974 | -49.58 to 39.63 | .788 |
| PML right  lateral-vermal | 10% | 3.062 | -13.92 to 20.04 | .690 |
|  | 20% | 4.083 | -20.84 to 29.00 | .720 |
|  | 30% | 20.90 | -5.340 to 47.14 | .102 |
|  | 40% | 0.7968 | -11.97 to 13.56 | .892 |
|  | 50% | 9.795 | -25.07 to 44.66 | .539 |
|  | 60% | 14.18 | -8.785 to 37.15 | .199 |
|  | 70% | -6.243 | -24.90 to 12.42 | .468 |
|  | 80% | 8.101 | -14.09 to 30.29 | .433 |
|  | 90% | 8.494 | -11.98 to 28.97 | .358 |
|  | 100% | 2.457 | -12.92 to 17.83 | .726 |
| CRUS II right  lateral-vermal | 10% | 16.78 | -23.19 to 56.76 | .369 |
|  | 20% | 34.61 | -3.702 to 72.93 | .072 |
|  | 30% | 28.00 | -15.52 to 71.53 | .179 |
|  | 40% | 31.01 | -7.505 to 69.52 | .103 |
|  | 50% | 13.79 | -33.98 to 61.56 | .529 |
|  | 60% | 4.344 | -39.56 to 48.25 | .823 |
|  | 70% | -4.618 | -25.55 to 16.31 | .632 |
|  | 80% | 6.914 | -12.22 to 26.05 | .439 |
|  | 90% | 10.71 | -4.666 to 26.09 | .150 |
|  | 100% | 11.01 | -3.498 to 25.51 | .118 |
| CRUS I right  lateral-vermal | 10% | -69.85 | -253.2 to 113.5 | .356 |
|  | 20% | -23.56 | -231.3 to 184.2 | .781 |
|  | 30% | -80.94 | -284.8 to 122.9 | .370 |
|  | 40% | -34.82 | -129.2 to 59.59 | .423 |
|  | 50% | -90.13 | -272.8 to 92.49 | .256 |
|  | 60% | -54.03 | -242.2 to 134.1 | .492 |
|  | 70% | -5.838 | -72.30 to 60.62 | .833 |
|  | 80% | -16.03 | -56.44 to 24.37 | .392 |
|  | 90% | 2.050 | -30.70 to 34.80 | .891 |
|  | 100% | -4.424 | -32.92 to 24.07 | .730 |
| LS right  lateral-vermal | 10% | 23.54 | -34.51 to 81.58 | .373 |
|  | 20% | 7.471 | -49.84 to 64.79 | .777 |
|  | 30% | 27.78 | -42.37 to 97.94 | .397 |
|  | 40% | 26.87 | -65.50 to 119.2 | .531 |
|  | 50% | -21.82 | -103.9 to 60.30 | .546 |
|  | 60% | 37.28 | -45.20 to 119.8 | .334 |
|  | 70% | 26.98 | -29.00 to 82.96 | .308 |
|  | 80% | 55.71 | -28.71 to 140.1 | .169 |
|  | 90% | 27.43 | -26.52 to 81.38 | .276 |
|  | 100% | -8.962 | -46.27 to 28.35 | .590 |
| X  right-vermal-left | 10% | -29.41 | -63.40 to 4.578 | .079 |
|  | 20% | 10.41 | -12.50 to 33.32 | .334 |
|  | 30% | -9.336 | -47.37 to 28.70 | .595 |
|  | 40% | -8.054 | -58.42 to 42.31 | .718 |
|  | 50% | -7.510 | -90.74 to 75.72 | .838 |
|  | 60% | 5.956 | -85.03 to 96.94 | .887 |
|  | 70% | 5.414 | -65.29 to 76.12 | .857 |
|  | 80% | -7.903 | -77.92 to 62.11 | .789 |
|  | 90% | 0.4477 | -24.66 to 25.55 | .967 |
|  | 100% | -3.182 | -43.59 to 37.23 | .863 |
| IX  right-vermal-left | 10% | 0.9931 | -20.16 to 22.15 | .919 |
|  | 20% | -1.526 | -56.11 to 53.06 | .949 |
|  | 30% | -13.59 | -62.84 to 35.67 | .551 |
|  | 40% | -10.15 | -94.87 to 74.57 | .794 |
|  | 50% | -15.21 | -99.30 to 68.88 | .690 |
|  | 60% | -4.662 | -78.10 to 68.78 | .890 |
|  | 70% | -38.58 | -101.4 to 24.25 | .201 |
|  | 80% | 24.95 | -29.56 to 79.47 | .332 |
|  | 90% | -10.16 | -45.65 to 25.32 | .537 |
|  | 100% | 18.76 | -35.83 to 73.36 | .455 |
| VIII  right-vermal-left | 10% | -5.890 | -22.20 to 10.42 | .435 |
|  | 20% | -7.932 | -38.20 to 22.33 | .570 |
|  | 30% | -9.074 | -50.95 to 32.81 | .639 |
|  | 40% | 16.89 | -23.64 to 57.42 | .371 |
|  | 50% | -13.51 | -58.17 to 31.14 | .512 |
|  | 60% | 22.10 | -26.45 to 70.65 | .332 |
|  | 70% | -4.583 | -45.54 to 36.37 | .808 |
|  | 80% | 0.9479 | -50.24 to 52.13 | .968 |
|  | 90% | 9.894 | -9.987 to 29.78 | .292 |
|  | 100% | 4.676 | -14.17 to 23.52 | .592 |
| VII  right-vermal-left | 10% | 10.92 | 0.8168 to 21.02 | .**037** |
|  | 20% | 3.060 | -6.254 to 12.37 | .481 |
|  | 30% | 2.141 | -2.341 to 6.623 | .312 |
|  | 40% | -0.8157 | -6.755 to 5.124 | .736 |
|  | 50% | -0.1243 | -5.841 to 5.593 | .962 |
|  | 60% | 3.232 | -6.871 to 13.34 | .492 |
|  | 70% | 4.324 | -5.805 to 14.45 | .348 |
|  | 80% | -0.04456 | -4.531 to 4.442 | .983 |
|  | 90% | 1.349 | -4.919 to 7.617 | .631 |
|  | 100% | -3.945 | -16.29 to 8.399 | .484 |
| VI  right-vermal-left | 10% | 4.143 | -30.64 to 38.93 | .775 |
|  | 20% | 11.01 | -44.14 to 66.15 | .637 |
|  | 30% | 14.76 | -33.03 to 62.55 | .505 |
|  | 40% | 10.66 | -12.69 to 34.01 | .332 |
|  | 50% | -13.49 | -47.25 to 20.27 | .378 |
|  | 60% | 17.48 | -15.03 to 49.98 | .256 |
|  | 70% | 27.33 | -24.85 to 79.50 | .255 |
|  | 80% | -0.2498 | -49.82 to 49.32 | .991 |
|  | 90% | 8.320 | -36.44 to 53.08 | .679 |
|  | 100% | 17.69 | -21.04 to 56.42 | .332 |
| IV/ V  right-vermal-left | 10% | 17.84 | -41.78 to 77.46 | .516 |
|  | 20% | 10.38 | -65.17 to 85.92 | .764 |
|  | 30% | -21.82 | -57.09 to 13.45 | .189 |
|  | 40% | -46.39 | -128.0 to 35.27 | .213 |
|  | 50% | -32.19 | -105.6 to 41.23 | .349 |
|  | 60% | -23.31 | -72.84 to 26.22 | .312 |
|  | 70% | -23.92 | -103.4 to 55.56 | .517 |
|  | 80% | -0.9227 | -45.26 to 43.41 | .963 |
|  | 90% | 17.68 | -45.27 to 80.63 | .545 |
|  | 100% | 51.30 | -16.42 to 119.0 | .120 |
| III  right-vermal-left | 10% | 3.959 | -19.71 to 27.63 | .716 |
|  | 20% | 4.592 | -12.14 to 21.33 | .551 |
|  | 30% | -16.98 | -42.82 to 8.848 | .174 |
|  | 40% | -20.13 | -80.34 to 40.09 | .473 |
|  | 50% | -28.45 | -132.9 to 76.01 | .545 |
|  | 60% | 9.632 | -93.08 to 112.3 | .838 |
|  | 70% | 1.728 | -83.58 to 87.04 | .964 |
|  | 80% | 1.620 | -34.62 to 37.86 | .921 |
|  | 90% | 8.337 | -9.721 to 26.39 | .327 |
|  | 100% | 8.892 | -14.01 to 31.79 | .395 |
| II  right-vermal-left | 10% | -2.052 | -28.43 to 24.32 | .854 |
|  | 20% | -20.28 | -62.02 to 21.46 | .291 |
|  | 30% | -13.28 | -55.98 to 29.43 | .503 |
|  | 40% | -11.37 | -62.42 to 39.68 | .628 |
|  | 50% | -1.842 | -89.49 to 85.81 | .963 |
|  | 60% | -5.826 | -61.86 to 50.21 | .820 |
|  | 70% | -3.382 | -59.23 to 52.47 | .895 |
|  | 80% | -12.70 | -58.17 to 32.77 | .547 |
|  | 90% | -6.248 | -37.58 to 25.08 | .655 |
|  | 100% | -2.855 | -24.02 to 18.31 | .770 |
| I  right-vermal-left | 10% | 19.50 | -5.116 to 44.11 | .101 |
|  | 20% | 17.11 | 2.996 to 31.21 | .**023** |
|  | 30% | -6.360 | -48.55 to 35.83 | .742 |
|  | 40% | -27.74 | -84.11 to 28.63 | .263 |
|  | 50% | -14.09 | -60.33 to 32.16 | .484 |
|  | 60% | 16.92 | -61.00 to 94.85 | .617 |
|  | 70% | -3.266 | -61.26 to 54.72 | .898 |
|  | 80% | -29.36 | -66.28 to 7.570 | .099 |
|  | 90% | -1.049 | -22.97 to 20.87 | .915 |
|  | 100% | -1.039 | -22.04 to 19.96 | .914 |
| PF left vermal-lateral | 10% | -67.83 | -195.6 to 59.97 | .256 |
|  | 20% | 31.77 | -87.41 to 151.0 | .566 |
|  | 30% | -2.173 | -122.0 to 117.7 | .968 |
|  | 40% | 4.237 | -52.90 to 61.37 | .870 |
|  | 50% | -3.015 | -105.5 to 99.49 | .947 |
|  | 60% | -33.10 | -155.8 to 89.64 | .531 |
|  | 70% | 45.97 | -39.70 to 131.6 | .254 |
|  | 80% | -7.153 | -82.89 to 68.58 | .831 |
|  | 90% | 70.18 | 14.66 to 125.7 | .**020** |
|  | 100% | 33.44 | -81.75 to 148.6 | .524 |
| FL left  vermal-lateral | 10% | -37.06 | -202.1 to 127.9 | .621 |
|  | 20% | -10.85 | -178.3 to 156.6 | .888 |
|  | 30% | 16.23 | -161.0 to 193.5 | .837 |
|  | 40% | -8.740 | -172.8 to 155.3 | .908 |
|  | 50% | -49.76 | -236.7 to 137.2 | .549 |
|  | 60% | -7.960 | -197.7 to 181.8 | .924 |
|  | 70% | 6.210 | -117.9 to 130.3 | .912 |
|  | 80% | 10.46 | -83.70 to 104.6 | .805 |
|  | 90% | 14.69 | -33.72 to 63.10 | .509 |
|  | 100% | 1.234 | -15.48 to 17.95 | .871 |
| COP left vermal-lateral | 10% | -11.28 | -35.63 to 13.08 | .296 |
|  | 20% | -6.078 | -54.32 to 42.17 | .780 |
|  | 30% | 15.07 | -27.04 to 57.18 | .411 |
|  | 40% | -38.58 | -84.91 to 7.762 | .092 |
|  | 50% | 4.988 | -26.01 to 35.99 | .725 |
|  | 60% | 13.90 | -35.28 to 63.08 | .542 |
|  | 70% | 3.683 | -20.56 to 27.92 | .741 |
|  | 80% | 7.126 | -27.02 to 41.27 | .650 |
|  | 90% | 11.75 | -15.40 to 38.89 | .352 |
|  | 100% | 7.231 | -31.98 to 46.44 | .665 |
| PML left vermal-lateral | 10% | 1.755 | -12.51 to 16.02 | .784 |
|  | 20% | 5.554 | -14.35 to 25.46 | .528 |
|  | 30% | 4.679 | -29.42 to 38.77 | .741 |
|  | 40% | 16.20 | -10.48 to 42.87 | .206 |
|  | 50% | -21.11 | -65.89 to 23.67 | .295 |
|  | 60% | 6.110 | -20.17 to 32.39 | .615 |
|  | 70% | 0.9864 | -28.92 to 30.90 | .943 |
|  | 80% | 21.18 | -12.45 to 54.82 | .191 |
|  | 90% | 25.38 | -3.472 to 54.24 | .078 |
|  | 100% | 5.905 | -7.053 to 18.86 | .326 |
| CRUS II left vermal-lateral | 10% | -6.615 | -16.85 to 3.624 | .176 |
|  | 20% | 0.9407 | -11.46 to 13.34 | .869 |
|  | 30% | 11.01 | -3.045 to 25.06 | .110 |
|  | 40% | -24.86 | -52.33 to 2.606 | .068 |
|  | 50% | 21.84 | -27.11 to 70.80 | .322 |
|  | 60% | 31.27 | 1.638 to 60.91 | .**041** |
|  | 70% | 24.61 | -8.731 to 57.96 | .130 |
|  | 80% | 20.77 | 1.657 to 39.88 | .**036** |
|  | 90% | 51.26 | -0.2381 to 102.8 | .051 |
|  | 100% | 33.40 | -9.542 to 76.34 | .106 |
| CRUS I left vermal-lateral | 10% | -4.946 | -18.15 to 8.260 | .422 |
|  | 20% | -12.31 | -45.39 to 20.76 | .393 |
|  | 30% | -24.69 | -76.30 to 26.91 | .273 |
|  | 40% | -63.15 | -167.7 to 41.44 | .196 |
|  | 50% | -108.9 | -290.1 to 72.36 | .203 |
|  | 60% | -104.8 | -281.6 to 72.10 | .214 |
|  | 70% | 39.58 | -146.6 to 225.7 | .645 |
|  | 80% | 6.490 | -183.0 to 196.0 | .941 |
|  | 90% | 94.79 | -31.09 to 220.7 | .122 |
|  | 100% | 85.55 | -62.52 to 233.6 | .213 |
| LS left vermal-lateral | 10% | 6.730 | -23.09 to 36.55 | .625 |
|  | 20% | -11.02 | -64.19 to 42.15 | .639 |
|  | 30% | 9.331 | -77.09 to 95.75 | .814 |
|  | 40% | -10.03 | -96.40 to 76.33 | .800 |
|  | 50% | 45.92 | -34.35 to 126.2 | .231 |
|  | 60% | 94.98 | 2.757 to 187.2 | .**045** |
|  | 70% | 90.58 | 7.549 to 173.6 | .**035** |
|  | 80% | 76.88 | -9.822 to 163.6 | .076 |
|  | 90% | 36.97 | -31.60 to 105.5 | .256 |
|  | 100% | 33.49 | -13.82 to 80.80 | .145 |

Supplementary Table 7: hemispheric differences in PC activity in sublobular areas

| **Early extinction multiple paired t test hemispheric difference of normalized PC** | | | | |  |
| --- | --- | --- | --- | --- | --- |
| **lobule** | **bin of lobule** | **mean diff.** | **t ratio** | **p-value** | |
| Pf right vs left | Bin 2 | -104 | 5.1 | **.004** | |
| CRUS II right vs left | Bin 2 | -11 | 0.71 | .509 | |
|  | Bin 4 | 6.4 | 0.48 | .646 | |
| LS right vs left |  | -10 | 0.52 | .621 | |
|  | Bin 4 | -64 | 1.5 | .194 | |
|  | Bin 5 | -117 | 3.3 | **.016** | |
| VII right vs left | Bin 1 | 2.3 | -3.9 | .059 | |
| VI right vs left | Bin 2 | -1.0 | 2.0 | .093 | |
|  |  |  |  |  | |

**References**

Batsikadze, G., Diekmann, N., Ernst, T. M., Klein, M., Maderwald, S., Deuschl, C., et al. (2022). The cerebellum contributes to context-effects during fear extinction learning: A 7T fMRI study. *Neuroimage* 253, 119080. doi: 10.1016/j.neuroimage.2022.119080

Batsikadze, G., Pakusch, J., Klein, M., Ernst, T. M., Thieme, A., Nicksirat, S. A., et al. (2024). Mild Deficits in Fear Learning: Evidence from Humans and Mice with Cerebellar Cortical Degeneration. *eNeuro* 11, ENEURO.0365-23.2023. doi: 10.1523/eneuro.0365-23.2023

Ernst, T. M., Brol, A. E., Gratz, M., Ritter, C., Bingel, U., Schlamann, M., et al. (2019). The cerebellum is involved in processing of predictions and prediction errors in a fear conditioning paradigm. *Elife* 8. doi: 10.7554/elife.46831

Nio, E., Pereira, P. P., Diekmann, N., Petrenko, M., Doubliez, A., Ernst, T. M., et al. (2025). Human cerebellum and ventral tegmental area interact during extinction of learned fear. doi: 10.7554/eLife.105399.1

Schindelin, J., Arganda-Carreras, I., Frise, E., Kaynig, V., Longair, M., Pietzsch, T., et al. (2012). Fiji: an open-source platform for biological-image analysis. *Nature Methods 2012 9:7* 9, 676–682. doi: 10.1038/nmeth.2019
